# Polycystic Ovarian Syndrome: An Autobiographical Case Report of an Often Overlooked Disorder

**DOI:** 10.7759/cureus.21171

**Published:** 2022-01-12

**Authors:** Vithi Hitendra Patel

**Affiliations:** 1 Family Medicine, Gujarat Medical Education & Research Society (GMERS) Medical College and Hospital, Valsad, IND; 2 Internal Medicine, Gujarat Cancer Society Medical College and Research Center, Ahmedabad, IND

**Keywords:** management, lifestyle intervention, diet and exercise, diagnosis, insulin resistance, psychosocial impact, quality of life, pcos

## Abstract

Polycystic ovary syndrome (PCOS) is a highly prevalent disease seen in women of reproductive age, and yet a majority of cases go undiagnosed. This autobiographical case report describes a young doctor's experience with PCOS and attempts to highlight the significance of a missed diagnosis. In addition to the endocrine system, PCOS affects the metabolic, reproductive, mental, and psychosocial health of women. Manifested symptoms are just the tip of the iceberg, and addressing what's underneath is the real challenge. The disease in its untreated and undiagnosed forms leads to a series of co-morbidities including, but not limited to, obesity, infertility, diabetes mellitus, cardiovascular disease, and cancer. Additionally, the emotional burden in PCOS women is a major threat to their quality of life and needs separate acknowledgment. PCOS is a pressing issue; the long-term consequences on physical and mental health need to be taken seriously.

## Introduction

Polycystic ovary syndrome (PCOS) was originally delineated in 1935 by Leventhal and Stein. According to the Rotterdam consensus, PCOS diagnosis should be based on the presence of any two of the following three criteria: (a) irregular menstruation i.e. oligomenorrhea and/or anovulation, (b) clinical and/or biochemical evidence of hyperandrogenism, and (c) ultra-sonographic evidence of polycystic ovaries [1]. It is a hyperandrogenic disorder with an approximate prevalence of 15% to 20%; despite being a common disease in women, an estimated 68% of the total cases remain undiagnosed [2]. At least a third of women reported visiting multiple clinicians before a diagnosis is established [3].

Late diagnosis and no timely measures increase the risk of progressing to avoidable, adverse consequences associated with the syndrome. As many as 30% of adolescent females with PCOS are at risk of metabolic syndrome [4]. Another study estimates around 40% of females with PCOS would suffer from diabetes mellitus by their 50's [5]. Women with PCOS have three-to-four-fold times increased risk of developing early-onset endometrial cancer [6]. In addition to the physical concerns, women with PCOS have eminent physiological distress. A Brazilian research study concluded that 58% of women with PCOS exhibited at least one psychiatric disorder [7]. 

Long-term consequences of PCOS on psychological health have been underestimated and disregarded [8]. Even though the disorder has a wide spectrum of symptoms affecting a female's life from early teenage to later in life, it is not given as much importance as other chronic diseases like diabetes mellitus, hypertension, or a thyroid disorder. The need to address this syndrome both as a burden to the healthcare system, as well as on an individual basis right now, is of paramount importance.

The purpose of this case report is to highlight the need for an early diagnosis, the emotional and psychological impact of the disease, and the need for a multi-disciplinary approach to improve the quality of life and prevent associated comorbidities.

## Case presentation

I had been struggling with acne since my teenage years and consulted multiple doctors for the same. During my medical school days, I was very satisfied with the results after consulting a renowned dermatologist. I was diagnosed with acne vulgaris and was prescribed multiple oral antibiotics ranging from penicillins to linezolid along with oral contraceptive (OC) pills and isotretinoin. I would frequently visit him every 10-15 days for almost one and a half years. I was, and still am, so grateful to my provider for having successfully dealt with my acne. Despite that, I could not help but feel that my treatment was only skin-deep. As a medical intern, I started having my suspicions that I might have PCOS based on the symptoms I was experiencing. The first severe PCOS flare-up was when we were posted in the labor room for our obstetrics and gynecology (OBGYN) rotations. I put on almost five kilograms to my weight over the period of a month. It felt like my clothes started to fit differently almost overnight. Unlike the usual teenage acne that I had before, the new ones were the cystic kind being more severe and painful. Even a slight touch to it or light face-washing would cause unbearable pain. These manifestations wreaked havoc on the psychosocial aspects of my life. When I would bring up my concerns to my primary care physician, they would be dismissed without a proper investigation. The following year, I was also diagnosed with generalized anxiety. None of these indicators were appropriately considered for a PCOS diagnosis, and it took until almost two years later when I had continuous menstrual bleeding for almost 45 days. Morphological complaints and poor quality of life were not given enough merits to be investigated. The late diagnosis took its toll, not only on my physical well-being but on my professional and personal life as well. I was working as a junior resident in the medicine department, which made long shifts much more difficult. Working with personal protective equipment (PPE) in a COVID-19 ward was a whole other issue. I was only criticized, not supported, for bringing up the challenges that PCOS brought to my work life. Growing up, I used to be confident in my abilities, but the disease, in its undiagnosed form, pushed me into seclusion. I became more and more diffident and started avoiding going out in public and socializing less in general. The two years before my diagnosis and the time it took to cope after were probably the hardest in my life. 

Symptoms

Irregular Menstrual Cycles

As a result of ovulatory dysfunction, irregular menstrual cycles are a key symptom of PCOS. What makes it harder to identify as a symptom of PCOS is that they are not uncommon during puberty. In my case, I had a very regular cycle which was probably part of the reason for the late diagnosis. In turn, I had to deal with the anxiety, stress, and frustration further in the future because the disease had then progressed far enough to cause abnormal uterine bleeding for almost two months. 

Acne

Acne is common in teenagers due to a physiological increase in androgen levels during puberty. However, treatment-resistant acne should be considered as a sign of hyperandrogenism, and more importantly, should be investigated further [9]. In my case, acne was not simply another symptom; it affected more than my appearance. The cystic acne would get so bad that social embarrassment and withdrawal had become greater issues than the excruciating pain that they brought with them. Social events should be fun and relaxing; however, my acne would always be a topic of discussion, and it would only add to my feelings of self-deprecation. 

Hirsutism

Hirsutism is one of the major contributing factors to PCOS's psychological distress [10]. I have had it since adolescence, but more recently, it has increased and has become more prominent. Not only is its appearance distressing, but managing it takes considerable time and energy on my part. 

Obesity

Central obesity is one of the major components of metabolic syndrome, along with insulin resistance, hypertension, and dyslipidemia. Women with PCOS have a higher risk of metabolic syndrome and its cardiovascular sequelae [11]. Similar to other PCOS patients, I swiftly gained a good amount of weight. It went unrecognized as a symptom of PCOS due to a difficult phase in my life that represented a lot of unhealthy eating and a little-to-no exercise routine. Unfortunately, losing excess weight in PCOS is easier said than done, and I would only find that out after making numerous failed attempts. Initially, the only complaint I had with this was not being able to fit in my favorite denim. But soon after, I realized I was having uncontrolled sugar cravings, increased urinary frequency, and mood swings, all symptoms consistent with an impaired glucose tolerance, which is frequently seen in diabetes mellitus patients. My fasting glucose levels were normal, but I had increased fasting insulin and postprandial insulin levels, which would indicate the possibility of having diabetes mellitus in the future. In spite of being aware of that, sometimes the sugar cravings would worsen to such an extent that I would feel unable to function without satisfying it, and doing so, in turn, would have a paradoxical effect on my mood. 

Psychological Distress

The association between psychological distress and PCOS is well documented [12]. The symptoms of PCOS and stress are a part of a vicious cycle. For me, stressful events such as examinations, night-call duties would lead to a flare-up of symptoms, which in turn only added to the stress. 

Dealing with study, social life, and family issues was hard enough without suffering from a debilitating disease, and together, it led to poor sleep. I was diagnosed with an anxiety-induced sleep disorder and was prescribed lorazepam for the same. I used to be a social butterfly, but PCOS affected my self-esteem to such a degree that I would avoid even casual meet-ups, and anxiety filled every corner of my being. Symptoms like acne, hirsutism, and obesity had a powerful, negative impact on the psychosocial aspects of life. 

An additional anguishing part of the disease was lethargy. I remember feeling tired all the time; my to-do list only kept increasing, and I would never be able to get around to finishing any of it. Even small tasks would require a lot of mental preparation and self-persuasion. 

Diagnosis

As mentioned earlier, a good proportion of women with PCOS remain undiagnosed. The disease is brought to attention only when complications surface. For me, having been diagnosed late is highly regrettable, and it took a sizable toll that I hope other women do not have to pay. My complaints of acne, weight gain, and psychological distress were considered too inadequate to warrant the need for investigations. By the time menstrual irregularities set in, my OBGYN doctor and I were quite sure of PCOS being the probable diagnosis, but we were waiting for the investigations. The clinical diagnosis was verified with an abdominal ultrasound showing more than 15 small follicles in both the ovaries and increased ovarian volume to 11 ccs with a biochemical investigation of hyperandrogenic state. Even while having an idea of the probable diagnosis, the confirmation of the same was quite difficult to come to terms with. 

Treatment

PCOS neither has a definite cure nor treatment. It is challenging because the disease has no universal treatment applicable to every woman. Management fundamentally focuses on treating manifested symptoms and irregularities in investigations. But based on my experience with the disease, I would like to highlight the need of incorporating a holistic approach. Medicines and lifestyle changes are not enough to keep the disease in check. A little empathy and acknowledgment of the problem would go a long way.

Lifestyle Modifications

Weight loss is the best therapy to address hyperinsulinemia and hyperandrogenism symptoms seen in PCOS [13]. Weight loss is an effective antidote to chronic anovulation seen in PCOS. As my struggle was both physical and mental, I often lacked proper action during my weight loss journey. It is also well documented that losing weight is not easy for PCOS women [14]. I began working on my condition by adopting a vigorous exercise regimen, but it only added to the stress and hormonal imbalance. This made me realize the importance of taking things slow and the need for devising a comprehensive treatment plan with my doctors that worked best for my profile.

I actively participated in the treatment process by meticulously researching the latest literature online. I started keeping a diary of symptoms to get a better picture of the disease. The primary lifestyle modification revolved around regular and limited physical activity in the form of brisk walking and resistance training, alongside adherence to healthy food. However, despite knowing what I needed to do as a patient, from my training as a physician, only knowledge was insufficient to spur me to follow through. 

Working on my diet was also an excruciating part of my PCOS journey. As someone who has quite the sweet tooth and craves bread too, the diagnosis was devastating. Additionally, rice, wheat, and dairy are among the staple foods for Indians. In my case, the investigations of baseline insulin suggested some degree of insulin resistance, so a carbohydrate-based diet was deemed unideal. Therefore, restricting high-glycemic-index foods such as white bread, pasta, and rice was essential.

I was recommended a high-protein and low-carbohydrate diet. Being a vegetarian, I started by consuming more legumes. Additionally, certain foods are well established to induce inflammation in PCOS and worsen the condition [15]. I understood that no food could be entirely bad, but I would need to assess my diet and avoid offending foods. Managing a diet plan is very complex, which made me value the role of a nutritionist as a part of the care team. I do have my share of stressful days, where it is easier to give in to unhealthy food cravings, and that only ends up hindering my overall progress. However, after noticing the positive effects of changes to my diet, achieving my target weight seems within reach.

Both doctors and PCOS patients need to understand that while PCOS directly affects physical health, it comes with its own set of mental challenges for the patient. Hence, a multidisciplinary approach including diet, exercise, and stress management is crucial. Getting medical help from a nutritional counselor and psychiatrist should be an integral part of management in addition to the primary treating physician/OBGYN.

Medications

OC pills are used for controlling hyperandrogenemia. In my case, the course was first prescribed in 2013 by my dermatologist for acne vulgaris; the second time was in 2020 after the diagnosis of PCOS. My only prior exposure to OC pills was from the hesitancy that I had seen towards it from friends and family for reasons unknown to me. I, however, am privileged to have studied medicine to know that chronic use of OC pills for three to six months would mitigate an irregular menstrual cycle, as it did for me. This made me realize that proper patient counseling is required for good adherence to the devised management plan for achieving favorable results. On the other hand, indefinite reliance on hormonal pills to keep symptoms under check should not be advisable as they have recurrent drug interactions and nasty side effects [16]. Rather than simply providing treatment for the presenting symptoms, a thorough investigation must be prioritized. In my case, a timely diagnosis would have helped me better manage the condition before the complications set in. 

Metformin works on all the parameters of the PCOS-associated metabolic syndrome. It is the drug of choice for correcting insulin resistance. Unfortunately, I had already developed abdominal obesity by the time I was diagnosed with PCOS. I had been prescribed metformin 500 mg with 200mg alpha-lipoic acid twice a day. My initial experience of taking metformin was not pleasant; I developed a severe loss of appetite, nausea, drowsiness, and lethargy. I will make no excuses for it, but I had temporarily stopped the medication without consulting my OBGYN doctor due to the side effects. Having experienced the other side of the table, I now have a better understanding of why it can be difficult for patients to follow their physician's instructions. Therefore a regular follow-up and a healthy patient-doctor relationship is a must to ascertain proper treatment.

Additionally, investigations suggested mild hyperprolactinemia, for which cabergoline 0.5 mg twice daily was added to my treatment plan. I was also prescribed tranexamic acid 250 mg with ethamsylate 250 mg for abnormal uterine bleeding. An important issue that I encountered was managing the timely intake of multiple pills; OC pills, metformin, and cabergoline are drugs that have rather strict timings. Add to that multiple lifestyle modifications, and that ensured that I found this whole situation very overwhelming. As some time passed, symptoms reduced or were resolved, and the dosage and medications were reduced. After much strife and struggle, I have only recently managed to reconcile myself to the diagnosis, including the complex lifestyle changes and medications. 

## Discussion

Undiagnosed, PCOS severely impacted my physical and mental well-being. Each physical symptom had associated psychological distress, which was overlooked. Lack of acknowledgment from family and friends added to it as well. While I understand the rationale for a delayed diagnosis being due to similarities between the PCOS symptoms and physiological puberty, there were multiple indicators that could have helped make the diagnosis earlier, like treatment-resistant acne, hirsutism, and obesity. The compilation of these symptoms could suggest an underlying disease. My experience as a PCOS patient with the diagnosis and treatment was unsatisfactory. I felt that more could and should have been done to evaluate my distress even though my doctor treated the disease well as per the protocol. The constellation of the presenting symptoms should raise suspicions for PCOS, and symptomatic prescription should not be practiced without thorough investigations. I felt a lack of proper counseling and frequent follow-ups to monitor my progress. The early inception of a team consisting of a nutritionist, and an endocrinologist, could have been more beneficial.

The purpose of this autobiographical case report is to urge clinicians to have a high degree of clinical suspicion for PCOS to facilitate timely interventions and reduce the significant co-morbidities associated with it. I also tried to highlight the PCOS symptoms beyond their physical manifestations and propose the need to treat psychological distress as a part of syndrome management.

Clinicians often tend to overlook emotional stress linked with the disease. Women have reported negligence on the part of health care professionals for dismissing their concerns without sincere considerations [17]. As a healthcare professional, I can vouch that physicians intend to provide the best care for their patients; however, where they tend to fall short is when adopting a holistic approach. In my opinion, PCOS is one such condition where providing symptomatic care is not enough. It is vital that PCOS is not dismissed as a lifestyle disorder. An understanding and supportive response from the doctors not only attenuate the trepidation of diagnosis but also provide a positive outlook in managing the condition.

Healthcare providers need to build a trustful relationship with patients and be more compassionate about patients' concerns. The treating doctor should ascertain that the patient has fully understood the disease. It has been established that weight loss is the best therapy to prevent metabolic complications and also improve the quality of life [18]. Therefore, making people understand the importance of weight loss would likely yield a better result than simply advising the patient to lose weight; active participation on the patient's part is essential. Routine and frequent follow-ups are vital to monitoring the progress. Positive outcomes in controlling the symptoms and averting the risk of metabolic syndrome can only be achieved with contributions from both patients and physicians.

Another management strategy would involve individualizing the treatment plans. PCOS is a complex disorder, and therapy needs to be curated according to the patient's profile. What works for someone might not for others. A group of multiple specialists needs to be involved as a part of standard care in PCOS. 

## Conclusions

PCOS is a chronic disease affecting women's health throughout their lives. The patient's womanhood may be challenged by acne, hirsutism, obesity, and sub-fertility brought by the disease. In addition, the condition is plagued by delayed diagnosis, poor quality of life, and substandard management. The simplest solution to this problem is to acknowledge the complexity of the disease and individualize the treatment process to each patient with the involvement of a team of specialists. To ensure quality care, psychological distress also needs to be addressed as a part of the management. In addition to medical treatment, support from family and friends, good nutrition, physical activity, disease ownership, and personal advocacy can enable thriving despite the disease.
